# Annual acknowledgement of manuscript reviewers

**DOI:** 10.1186/2049-1891-5-10

**Published:** 2014-02-13

**Authors:** Defa Li

**Affiliations:** 1 China Agriculture University, Beijing, China

## Abstract

**Contributing reviewers:**

The editors of *Journal of Animal Science and Biotechnology* would like to thank all our reviewers who have contributed to the journal in Volume 4 (2013).

## 

Andre Aarnink

Netherlands

Hansjoerg Abel

Germany

Marieke Abrahamse-Berkeveld

Netherlands

Mohammad Al-Mamun

Bangladesh

Ousama AlZahal

Canada

Wageha Awad

Austria

Francis Barillet

France

Marc Bauer

United States of America

Fuller Bazer

United States of America

Donald Beitz

United States of America

Donagh Berry

Ireland

Shashi Bhushan

India

Mario Binelli

Brazil

Dustin Boler

United States of America

Paolo Bosi

Italy

Laura Brown

United States of America

Gerardo Caja

Spain

Laurianne Canario

France

Kathy Carlstead

United States of America

Margarida Castell

Spain

Gérard Chaouat

France

Farid Chemat

France

Bernadette Chen

United States of America

Beth Cleveland

United States of America

Joe Crenshaw

United States of America

Monica Isabella Cutrignelli

Italy

Dirk Dannenberger

Germany

David Davies

United Kingdom

Stefaan De Smet

Belgium

Jared Decker

United States of America

Elizabeth DeLyria

United States of America

Elitsa Dimova

Finland

Hua Ding

United States of America

Sami Dridi

United States of America

Tom Druet

Belgium

Mike Dugan

Canada

Samer El-Kadi

United States of America

Mauricio Elzo

United States of America

Chantal Farmer

Canada

Catherine Field

Canada

Guo-Hua Fong

United States of America

Tore Framstad

Norway

Shaorong Gao

China

Miriam Garcia

United States of America

Dorian Garrick

United States of America

Rodney Geisert

United States of America

Ailian Geng

China

Joshua Gong

Canada

Robert Goodband

United States of America

Rongchao Hao

China

Philip Harris

New Zealand

Brad Heins

United States of America

Kiichi Hirota

United Kingdom

Rhonda Hoffman

United States of America

Yongqing Hou

China

Clay Isom

United States of America

Breda Jakovac-Strajn

Slovenia

Peng Ji

United States of America

Runshen Jiang

China

Jennifer Johns

United States of America

Greg Johnson

United States of America

Cassandra Jones

United States of America

Henry Jorgensen

Denmark

Kampon Kaeoket

Thailand

Juha Kantanen

Finland

Sung Woo Kim

United States of America

Maarten Kootstra

Netherlands

Clint Krehbiel

United States of America

Rami Kridli

Jordan

Osman Kucuk

Turkey

Wan Sup Kwak

South Korea

Changhua Lai

China

Jean-Paul Lallès

France

Ting Lei

United States of America

April Leytem

United States of America

Changxi Li

Canada

Hai Lin

China

Jan Erik Lindberg

Sweden

Yanhong Liu

United States of America

Juan Loor

United States of America

Matthew Lucy

United States of America

Robert Maddock

United States of America

Hassan Malekinejad

Netherlands

Michelle Martinez-Montemayor

Puerto Rico

Francesca Martuzzi

Italy

Jon Meadus

Canada

Sam Millet

Belgium

Patrick Morel

New Zealand

Miquel Moretó

Spain

Ahmed Moussa

Algeria

Semone Myrie

Canada

Mark Nelson

United States of America

Anita Oberbauer

United States of America

Jack Odle

United States of America

Garrett Oetzel

United States of America

Jernej Ogorevc

Slovenia

Oluyinka Olukosi

United Kingdom

Suneel Onteru

India

Ameer Pahm

United States of America

Vishwanath Patil

Norway

Rosa Maria Pereira

Portugal

George Perry

United States of America

James Pettigrew

United States of America

John Pluske

Australia

Robert Possee

United Kingdom

Guanghai Qi

China

Srujana Rayalam

United States of America

Ana Isabel Rodrigues

Portugal

Albert Rosenberger

Germany

Rick Roush

Australia

Zemelak Sahle

Germany

Janeen Salak-Johnson

United States of America

James Sartin

United States of America

Chwan-Li (Leslie) Shen

United States of America

Paul Siegel

United States of America

Saulo Silva

Brazil

Fred Silversides

Canada

Leon Spicer

United States of America

Hans Stein

United States of America

Hui-Min Su

Taiwan

Lakshmi Sunkara

United States of America

Sylwester Swiatkiewicz

Poland

Sha Tao

United States of America

Anders Thygesen

Denmark

Yanan Tian

United States of America

Mike Tokach

United States of America

Manoj Kumar Tripathi

India

Swamy Tripurani

United States of America

Eleni Tsiplakou

Greece

Pedro Urriola

United States of America

Helena van Tol

Netherlands

Francielo Vendruscolo

Brazil

Martin Verstegen

Netherlands

Mirelle Viana

Brazil

Enrique Viturro

Germany

Elizabeth Walker

United States of America

Per Wallgren

Sweden

Chunkao Wang

United States of America

Xiaoqiu Wang

United States of America

Ying Wang

United States of America

Zurong Wang

Singapore

Malcolm Watford

United States of America

James Wells

United States of America

Kevin Wells

United States of America

De Wu

China

Ji Wu

China

Yang Xiaojian

Canada

Jingjing Xie

China

Wenzhi Xue

China

Ning Yang

China

Wenzhu Yang

Canada

Junhu Yao

China

Jingdong Yin

China

Yulong Yin

China

Curtis Youngs

United States of America

Wei Zhai

United States of America

Gui-xiang Zhang

China

Feng-Qi Zhao

United States of America

Zhihui Zhao

China

Shien Zhu

China

Wei-Yun Zhu

China

